# Supplementary material for: Computational conjugate adaptive optics microscopy for longitudinal through-skull imaging of cortical myelin
Source: Nat Commun. 2023 Jan 6;14:105. doi: 10.1038/s41467-022-35738-9 (PMC9823103; doi:10.1038/s41467-022-35738-9)
Supplement: Supplementary file 1 — Supplementary information [file 41467_2022_35738_MOESM1_ESM.pdf]

Computational conjugate adaptive optics microscopy for longitudinal through-skull imaging of cortical myelin

Yongwoo Kwon[1,2,+], Jin Hee Hong[1,2,+], Sungsam Kang[1,2,+], Hojun Lee[1,2], Yonghyeon Jo[1,2], Ki Hean Kim[3], Seokchan Yoon[1,2,4,*]and Wonshik Choi[1,2,*]

[1]*Center for Molecular Spectroscopy and Dynamics, Institute for Basic Science, Seoul 02841, Korea*
[2]*Department of Physics, Korea University, Seoul 02855, Korea*
[3]*Department of Mechanical Engineering, Pohang University of Science and Technology, Pohang 37673, Korea*
[4]*School of Biomedical Convergence Engineering, Pusan National University, Yangsan 50612, Korea*
[+]*These authors contributed equally to this work.*
[*]*e-mail: sc.yoon@pusan.ac.kr and wonshik@korea.ac.kr*

1. Experimental details

1.1 Full experimental setup layout

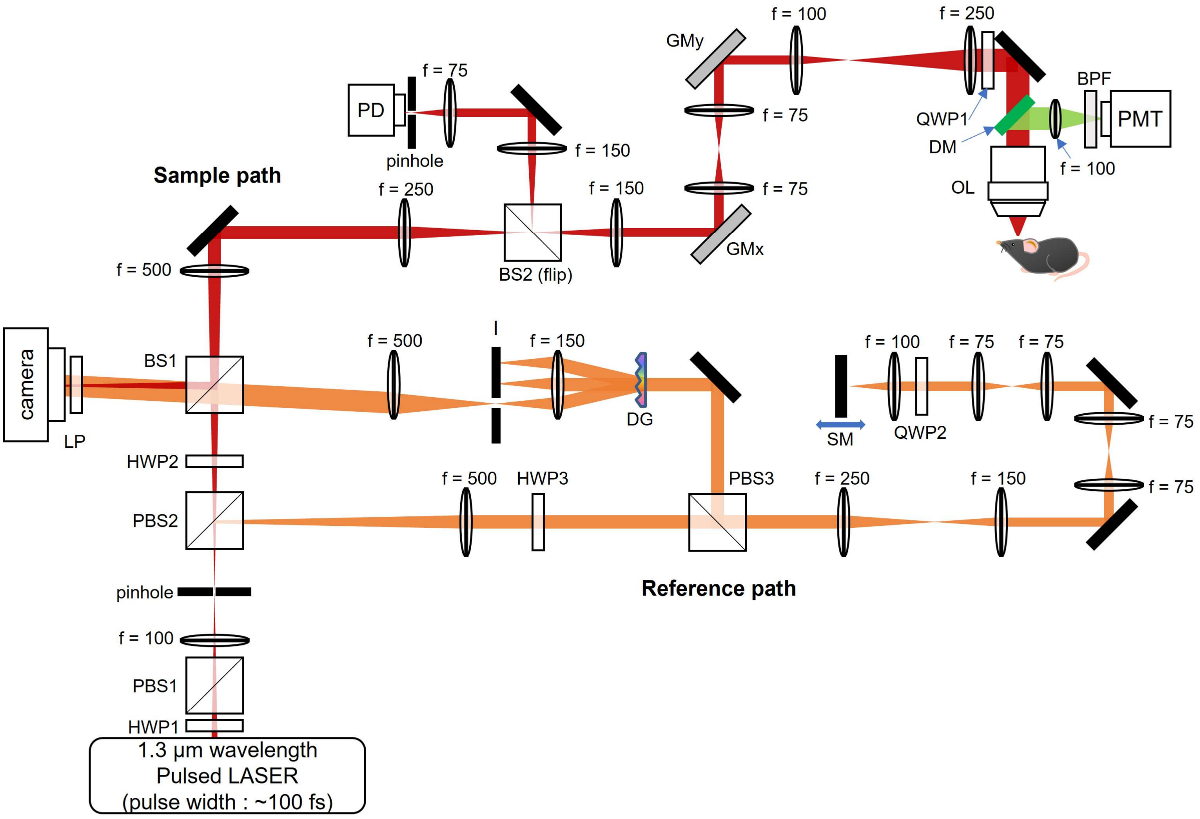

Supplementary Figure 1. Experimental schematic of 1.3 μm reflection matrix microscopy system. HWP: half-wave plate, PBS: polarizing beam splitter, BS: beam splitter (50:50), LP: linear polarizer, PD: photodetector, GMx and GMy: galvanometer mirrors for scanning a focused illumination along x and y directions, respectively, at the sample, QWP: quarter-wave plate, DM: dichroic mirror, BPF: band pass filter, PMT: photomultiplier tube, OL: objective lens, DG: diffraction grating, SM : scanning mirror, I: iris, f : focal length of lens in mm.

The backbone of the system is an interferometric confocal microscope, but we removed a confocal pinhole and measured phase and amplitude maps of all the elastic backscattering by using a camera. Wavelength-tunable pulsed laser (INSIGHT X3, Spectra physics) was used as a light source. Its bandwidth at the wavelength of 1.3 μm is 19 nm, which provides us with the coherence gating window (or time-gating window) of 25 μm. The output beam from the laser was delivered to the sample via two galvanometer scanning mirrors for the raster scanning of a focus illumination at the

sample plane. An objective lens (XLPLN25XWMP2, Olympus, 25x, 1.05 NA) with high NA and long working distance was used to ensure high spatial resolution deep within tissues. The backscattering signal from the sample was captured by the same objective lens and then descanned by the same scanning mirrors to be delivered to the camera. Fast InGaAs camera (Cheetah 800, Xenics, 6.8 kHz framerate) was placed at a plane conjugate to the sample to record interference image between the elastic backscattering from the sample and the reference beams. The off-axis interferogram was processed to obtain the phase and amplitude of sample waves for each illumination position.

The sample beam was delivered through 4-f configurations, and achromatic doublet lenses were used to minimize the chromatic and spherical aberration. Unlike our previous setup constructed at 900 nm, back-reflection noise from the various optical components was significantly large due to the relatively low quality of anti-reflection coating. A combination of a linear polarizer and a quarter-wave plate centered at 1310 nm was installed to remove the noise from the optics except the objective lens. Residual noise was further reduced via temporal gating set by the low-coherence interferometry. Also, the polarizing beamsplitters and half-wave plates were used for the control of input power and the power ratio between sample and reference path. The confocal reflectance image can be acquired both by camera and photodetector, but the photodetector is used to explore the sample over a large field of view. Our system can also record confocal fluorescence, second harmonic generation (SHG), and third harmonic generation (THG) images by installing a dichroic mirror right before the objective lens pupil along with PMT (H5784-20, Hamamatsu Photonics). The half-wave plate rotation stage for power control, the sample stage, galvanometer mirrors, reference path scanning mirror stage, data acquisition board, PMT, PD, and camera were controlled by MATLAB.

1.2 Data acquisition time

The acquisition time for each depth depends on the size of the region of interest (ROI). It takes 2.6 s for a ROI of 80×80 μm^2 and 10.4 s for 160×160 μm^2. The data acquisition time for the 3D imaging depends on both ROI and the number of depth acquisitions. The total acquisition time for each Supplementary Movie is given below.

Supplementary Movie 1: 160×160 μm^2 × 141 layers, 1466.4 s (24.4 min)
Supplementary Movie 2: 160×160 μm^2 × 146 layers, 1518.4 s (25.3 min)
Supplementary Movie 3: 160×160 μm^2 × 159 layers, 1653.6 s (27.6 min)

This volumetric image acquisition speed is good enough for monitoring the development of the myelination process occurring on a week-to-week time scale.

The through-skull imaging capability of our conjugate-CLASS comes from the acquisition of a reflection matrix, which requires the camera recording over a wide field of detection (FOD) for each focus scanning over the ROI. Therefore, the image acquisition speed is slower than that relying on the integral detection by a photodetector. However, we can speed up the image acquisition for a smaller ROI and FOD. For example, we can measure a reflection matrix for the ROI of 40×40 μm^2 in less than a second.

1.3 Decorrelation time due to the animal motion in the *in vivo* imaging

In our animal preparation procedure, a coverslip of 5 mm diameter was attached to the center of the parietal bone using an ultraviolet-curable glue. A custom-made metal plate was attached to the skull with cyanoacrylate for head fixation during the *in vivo* imaging, and the exposed part of the skull was covered with dental cement. Therefore, our system can be considered as a 'significantly immobilized' situation in the reference paper[1].

We measured the decorrelation time in the *in vivo* imaging. The acquisition time for a single-depth reflection matrix is as long as 10.4 s in our experiment. Therefore, we measured the image decorrelation for 30 s. Specifically, we illuminated the focused beam at a depth of 70 μm beneath the

dura and acquired a series of complex-field images over the field of detection of $75 \times 75\ \mu\text{m}^2$ at a frame rate of 2,000 Hz.

We calculated the intensity correlation r defined by the formula,

$$r = \frac{\sum_m \sum_n (A_{mn} - \bar{A})(B_{mn} - \bar{B})}{\sqrt{(\sum_m \sum_n (A_{mn} - \bar{A})^2)(\sum_m \sum_n (B_{mn} - \bar{B})^2))}} . \tag{S1}$$

Here A and B corresponds to the intensity of first image $I(t_0)$ and that of the image after time τ $I(t_0 + \tau)$, respectively, and $\bar{A}$ and $\bar{B}$ are their respective mean values. The m and n refer to the mth and n^{th} pixels, respectively, in each image. This formula is the same as the function $g_2(\tau)$ introduced in the reference paper[1]. Supplementary Fig. 2 shows that a high correlation is maintained during the time of 30 s with a minimum correlation value of 0.78. The regular peaks in the plot were due to the breathing of the mouse. The correlation value remained higher than 0.85 for the time duration of 10.4 s, which is the single-depth recording time for 160 μm^2 ×160 μm^2. This result supports that the tissue make little effect on our in vivo imaging.

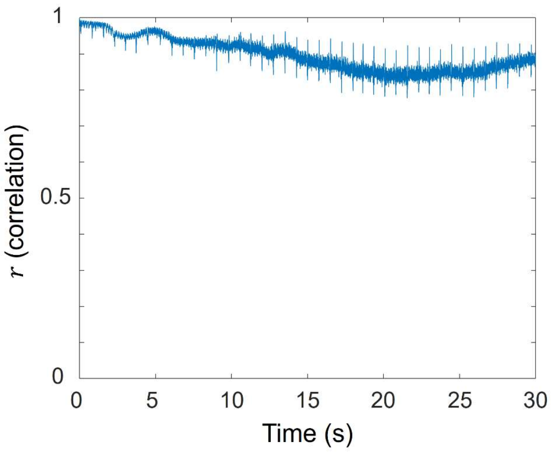

Supplementary Figure 2. Image correlation with time. The correlation r was calculated from a set of time-gated images acquired for 30 s while the focused illumination was parked at a depth of 70 μm beneath the dura. The correlation value stayed higher than 0.78 over the entire measured time span.

1.4 The choice of the subregion size

The major advantage of the conjugate AO over pupil AO in the through-skull imaging is its larger isoplanatic patch size. For the same experimental data, we found that the isoplanatic size of the pupil-CLASS was around $15 \times 15\ \mu\text{m}^2$ while that of our conjugate-CLASS was around $80 \times 80\ \mu\text{m}^2$ (Supplementary Fig. 8). Therefore, the size of each subregion to be analyzed can be as large as $80 \times 80\ \mu\text{m}^2$. Since the recorded ROI was $160 \times 160\ \mu\text{m}^2$, we can divide the entire ROI into 4×4 subregions with a 50 % overlapping ratio for optimal visibility. In this case, the computation time for reconstructing the entire ROI was 2,386 s. Note that the computation time is spent on the post-processing and, thus, does not affect to the in-vivo imaging. In the actual data analysis, we chose the subregion size of $64 \times 64\ \mu\text{m}^2$ and divided the entire ROI into 5×5 subregions, which resulted in a reduced computation time of 1557 s. In fact, as the patch size decreases and, thus, the number of patches increases, the total computation time gets shorter because the computation time for each patch scales with a power of 2 with respect to the area of the patch as shown in Supplementary Fig. 3. Since we processed the data over 140 to 170 depths in the volumetric image, this difference in computation time is a critical factor. We didn't reduce the subregion size to smaller than $64 \times 64\ \mu\text{m}^2$ to avoid image fragmentation in merging multiple images.

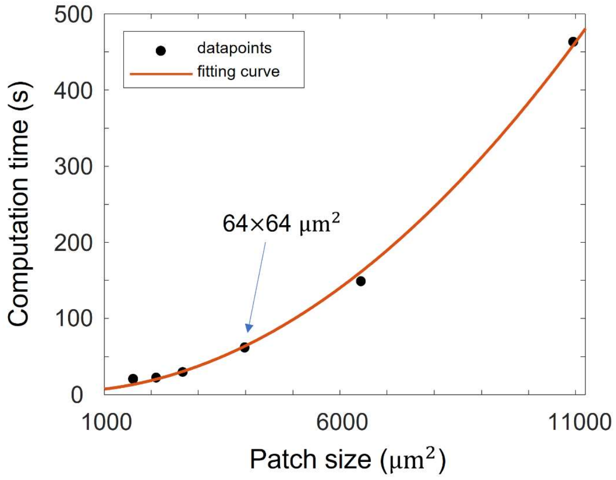

Supplementary Figure 3. Computation time depending on the analysis patch area. Data points in the figure correspond to 40×40 μm^2, 46×46 μm^2, 52×52 μm^2, 64×64 μm^2, 80×80 μm^2, and 105×105 μm^2. A computer used for this analysis is equipped with i9-12900KS CPU and NVIDIA RTX A6000 GPU. Note that the computation time for the full field of view is given by the multiplication of the computation time shown here to the number of patches. The data was well-fitted with the second-degree polynomial function.

1.5 Comparison between input and output aberration maps

The ϕ_{in} and ϕ_{o} should be the same due to the double-pass geometry and reciprocity. However, they can slightly be different due to experimental imperfections such as the slight misalignment between the illumination and detection pathways. Oftentimes, this misalignment is necessary to avoid direct back-reflection from the optics. In Fig. 2d, we showed only the output aberration maps as the input aberration maps are similar. Here we showed both the input and output aberration maps for a few different depths. The patterns have high similarity with their correlation value of 0.75 or higher.

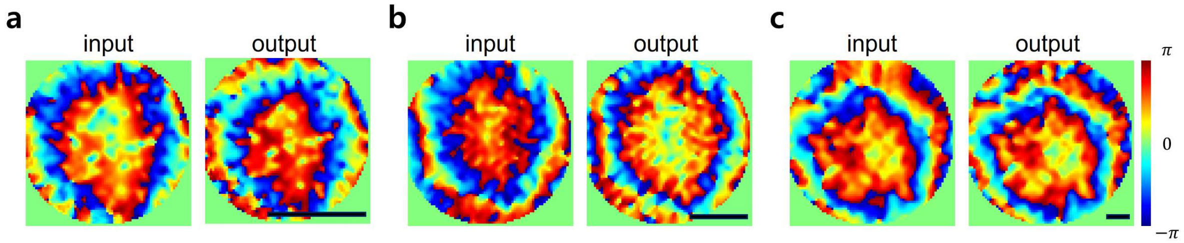

Supplementary Figure 4. Typical input and output aberration maps. a, Aberration maps at a depth of 60 μm beneath the dura. The correlation between the input and output aberration maps was 0.75. **b,** Same as **a**, but at a depth of 150 μm beneath the dura. Correlation value: 0.79. **c,** Same as **a**, but at a depth 450 μm beneath the dura. Correlation value: 0.80. Scale bars, 80 μm. Color bar, phase in radians.

1.6 Size of the aberration map in the conjugate-CLASS

The size of the scale bar in Fig. 2d varies with depth. This is because the size of the aberration map identified by the conjugate-CLASS increases with depth due to the illumination/detection geometry. As shown in Supplementary Fig. 5, the area in the skull plane responsible for the focused illumination is enlarged with the increase of the distance between the objective focus and the conjugate plane. The numerical aperture of the objective lens is another factor determining the aberration map size. In our experimental configuration, the diameter of the aberration map was given approximately by D~1.2z.

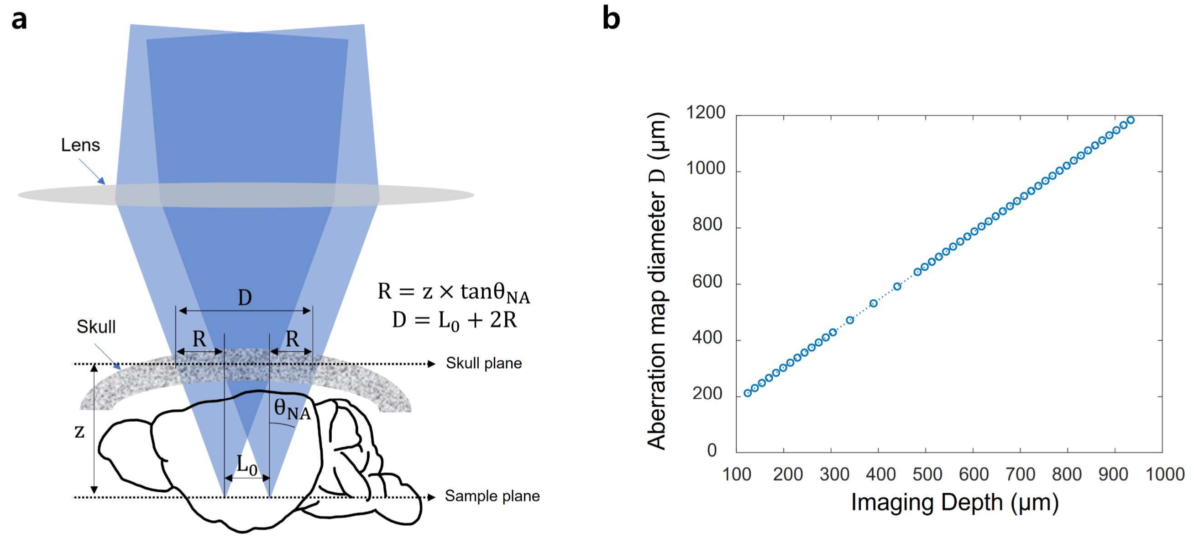

Supplementary Figure 5. The size of aberration maps at the skull plane. a, Schematic of two focused beams illuminating on the two ends of ROI at the sample plane through the skull. L_0: the size of ROI in one axis, which is typically 160 μm in the experiment. Θ_{NA}: the angle of the cone of the illumination beam. This can be calculated by $NA = n \times \sin\theta_{NA}$, where NA is the numerical aperture and n is the average refractive index of the medium. z: the distance from the sample plane to the skull plane. R: radius of the focused illumination in the skull plane, which can be calculated by $R = z \times \tan\theta_{NA}$. As a result, the diameter of aberration map D can be obtained by $D = L_0 + 2R$. **b,** The diameter of aberration map D depending on the imaging depth z. Relation between the diameter and the imaging depth was given by $D \sim 1.2z$.

2. Theoretical framework of the conjugate-CLASS

2.1. Basis conversion

The experimentally measured electric field $E(\mathbf{r}_\mathrm{o};\mathbf{r}_\mathrm{in})$ constituting $\boldsymbol{R}$ needs to be converted to $E(\mathbf{w}_\mathrm{o},\mathbf{w}_\mathrm{in})$, the matrix element of $\boldsymbol{R}_\mathrm{con}$ for the application of the conjugate-CLASS algorithm. This is realized by the free-space propagation of detected/illumination fields from $\mathbf{r}_\mathrm{o}/\mathbf{r}_\mathrm{in}$ to $\mathbf{w}_\mathrm{o}/\mathbf{w}_\mathrm{in}$. Let us suppose that the axial coordinate is $z=0$ for $\mathbf{r}_\mathrm{o}/\mathbf{r}_\mathrm{in}$, and that of $\mathbf{w}_\mathrm{o}/\mathbf{w}_\mathrm{in}$ is $z>0$. An incident wave focused at $\mathbf{w}_\mathrm{in}$ is a diverging spherical wave at $\mathbf{r}_\mathrm{in}$, which is expressed as $G(\mathbf{r}_\mathrm{in}-\mathbf{w}_\mathrm{in},z)$. Here $G(\mathbf{r},z)$ is the electric field of a point source emanating from the origin2:

$$G(\mathbf{r},z)=\frac{1}{i\lambda}\frac{\exp\left(in_0k\sqrt{|\mathbf{r}|^2+z^2}\right)}{\sqrt{|\mathbf{r}|^2+z^2}}\cdot\frac{z}{\sqrt{|\mathbf{r}|^2+z^2}}. \quad (S2)$$

Therefore, for the focused illumination at $\mathbf{w}_\mathrm{in}$, the electric field at $\mathbf{r}_\mathrm{o}$ is written as the superposition of $E(\mathbf{r}_\mathrm{o};\mathbf{r}_\mathrm{in})$:

$$E(\mathbf{r}_\mathrm{o};\mathbf{w}_\mathrm{in})=\int E(\mathbf{r}_\mathrm{o};\mathbf{r}_\mathrm{in})G(\mathbf{r}_\mathrm{in}-\mathbf{w}_\mathrm{in},z)\mathrm{d}^2\mathbf{r}_\mathrm{in}. \quad (S3)$$

The next step is to propagate the electric field measured at $\mathbf{r}_\mathrm{o}$ to $\mathbf{w}_\mathrm{o}$ located at -z in the reflection geometry. Since a point source emanating from $\mathbf{r}_\mathrm{o}$ is a diverging spherical wave $G(\mathbf{w}_\mathrm{o}-\mathbf{r}_\mathrm{o},z)$ at $\mathbf{w}_\mathrm{o}$, we can find the electric field at $\mathbf{w}_\mathrm{o}$ by the convolution of $E(\mathbf{r}_\mathrm{o};\mathbf{w}_\mathrm{in})$ with $G(\mathbf{w}_\mathrm{o}-\mathbf{r}_\mathrm{o},z)$:

$$E(\mathbf{w}_\mathrm{o};\mathbf{w}_\mathrm{in})=\int G(\mathbf{w}_\mathrm{o}-\mathbf{r}_\mathrm{o},z)\cdot E(\mathbf{r}_\mathrm{o};\mathbf{w}_\mathrm{in})\mathrm{d}^2\mathbf{r}_\mathrm{o}. \quad (S4)$$

We obtain $E(\mathbf{w}_\mathrm{o};\mathbf{w}_\mathrm{in})$ by the successive operations of Eq. (S2) and Eq. (S3) from $E(\mathbf{r}_\mathrm{o};\mathbf{r}_\mathrm{in})$.

2.2. Derivation of Eq. (1)

Figures 1b and 1c show the schematic configurations of the space-domain reflection matrix $\boldsymbol{R}$ and a conjugate-plane reflection matrix $\boldsymbol{R}_\mathrm{con}$, respectively. The electric field $E(\mathbf{w}_\mathrm{o};\mathbf{w}_\mathrm{in})$ constituting $\boldsymbol{R}_\mathrm{con}$ was described in Eq. (1) in the main text. Here, we present its detailed derivation.

A focused illumination at $\mathbf{w}_\mathrm{in}$ experiences phase retardation $\phi_\mathrm{S}(\mathbf{w}_\mathrm{in})$ by the skull and propagates to the sample plane at $\mathbf{r}=(x,y)$ at a distance z from the skull. Under Fresnel approximation, the electric field impinging on the sample plane is written as

$$E_\mathrm{in}(\mathbf{r};\mathbf{w}_\mathrm{in})=\frac{e^{ikz}}{i\lambda z}exp\left\{i\frac{k}{2z}|\mathbf{r}-\mathbf{w}_\mathrm{in}|^2\right\}e^{i\phi_\mathrm{S}(\mathbf{w}_\mathrm{in})}. \quad (S5)$$

The electric field reflected by the object at the sample plane is given by $E_\mathrm{r}(\mathbf{r};\mathbf{w}_\mathrm{in})=O(\mathbf{r})E_\mathrm{in}(\mathbf{r};\mathbf{w}_\mathrm{in})$, where $O(\mathbf{r})$ is the amplitude reflectance of the object at the sample plane. This reflected wave propagates back to the conjugate plane and experiences phase retardation $\phi_\mathrm{S}(\mathbf{w}_\mathrm{o})$ by the same skull. Therefore, the electric field of the backscattered wave at $\mathbf{w}_\mathrm{o}$ is expressed as

$$E(\mathbf{w}_\mathrm{o};\mathbf{w}_\mathrm{in})=\frac{e^{ikz}}{i\lambda z}e^{i\phi_\mathrm{S}(\mathbf{w}_\mathrm{o})}\iint E_\mathrm{r}(\mathbf{r};\mathbf{w}_\mathrm{in})exp\left\{i\frac{k}{2z}|\mathbf{w}_\mathrm{o}-\mathbf{r}|^2\right\}d^2\mathbf{r}. \quad (S6)$$

By inserting Eq. (S2) into Eq. (S3), we obtain $E(\mathbf{w}_\mathrm{o};\mathbf{w}_\mathrm{in})$ as

$$E(\mathbf{w}_\mathrm{o};\mathbf{w}_\mathrm{in})=-\frac{e^{2ikz}}{\lambda^2z^2}e^{i\phi_\mathrm{o}(\mathbf{w}_\mathrm{o})}\tilde{O}_\mathrm{M}\left(\frac{k}{z}(\mathbf{w}_\mathrm{o}+\mathbf{w}_\mathrm{in})\right)e^{i\phi_\mathrm{in}(\mathbf{w}_\mathrm{in})}. \quad (S7)$$

Here, $\phi_\mathrm{in}(\mathbf{w}_\mathrm{in})=\phi_\mathrm{S}(\mathbf{w}_\mathrm{in})+\frac{k}{2z}|\mathbf{w}_\mathrm{in}|^2$ and $\phi_\mathrm{o}(\mathbf{w}_\mathrm{o})=\phi_\mathrm{S}(\mathbf{w}_\mathrm{o})+\frac{k}{2z}|\mathbf{w}_\mathrm{o}|^2$. $\tilde{O}_\mathrm{M}$ is the 2D Fourier transform of $O_\mathrm{M}(\mathbf{r})=O(\mathbf{r})\exp\left\{i\frac{k}{z}|\mathbf{r}|^2\right\}$, where $O(\mathbf{r})$ is the amplitude reflectance of the target object at the sample plane. Since there exist multiple scattering backgrounds from the skull and brain tissues, the multiple-scattered wave $E_M(\mathbf{w}_\mathrm{o};\mathbf{w}_\mathrm{in})$ should be added to Eq. (S7), which is the Eq. (1) in the main text.

2.3. Conjugate-CLASS algorithm

We developed a conjugate-CLASS algorithm that finds $\phi_\mathrm{in}(\mathbf{w}_\mathrm{in})$, $\phi_\mathrm{o}(\mathbf{w}_\mathrm{o})$, and $\tilde{O}_\mathrm{M}$ from $E(\mathbf{w}_\mathrm{o};\mathbf{w}_\mathrm{in})$ in Eq. (1). Here, we exploit the fact that the object spectrum $\tilde{O}_\mathrm{M}(k(\mathbf{w}_\mathrm{o}+\mathbf{w}_\mathrm{in})/z)$ in $E(\mathbf{w}_\mathrm{o};\mathbf{w}_\mathrm{in})$ is shift-invariant with respect to $\mathbf{w}_\mathrm{in}$. Since this resembles the shift-invariance of $\tilde{O}(\mathbf{k}_\mathrm{o}-\mathbf{k}_\mathrm{in})$ with

respect to $\mathbf{k}_{\mathrm{in}}$ in the pupil-CLASS, we used a similar iterative optimization engine as that of the pupil-CLASS. The algorithm consists of two steps, first finding $\phi_{\mathrm{in}}(\mathbf{w}_{\mathrm{in}})$ and then $\phi_{\mathrm{o}}(\mathbf{w}_{\mathrm{o}})$.

2.3.1 Finding the approximate $\phi_{\mathrm{in}}(\mathbf{w}_{\mathrm{in}})$

We first replace $\mathbf{w}_{\mathrm{o}}$ with $\mathbf{W} = \mathbf{w}_{\mathrm{o}} + \mathbf{w}_{\mathrm{in}}$, which converts $E(\mathbf{w}_{\mathrm{o}}; \mathbf{w}_{\mathrm{in}})$ into

$$E(\mathbf{W}; \mathbf{w}_{\mathrm{in}}) = -\frac{e^{2ikz}}{\lambda^2 z^2} e^{i\phi_{\mathrm{o}}(\mathbf{W}-\mathbf{w}_{\mathrm{in}})} \tilde{O}_{\mathrm{M}}(k\mathbf{W}/z) e^{i\phi_{\mathrm{in}}(\mathbf{w}_{\mathrm{in}})} + E_M(\mathbf{W}; \mathbf{w}_{\mathrm{in}}). \quad \text{(S8)}$$

For each $\mathbf{w}_{\mathrm{in}}$, we compute the angle of the inner product $\langle E(\mathbf{W}; \mathbf{w}_{\mathrm{in}}) E^*(\mathbf{W}; \mathbf{w}_{\mathrm{in}} = 0)\rangle_{\mathbf{W}}$ to obtain the first estimate of $\phi_{\mathrm{in}}(\mathbf{w}_{\mathrm{in}})$:

$$\phi_{\mathrm{in}}^{(1)}(\mathbf{w}_{\mathrm{in}}) = \phi_{\mathrm{in}}(\mathbf{w}_{\mathrm{in}}) + \delta\phi_{\mathrm{in}}^{(1)}(\mathbf{w}_{\mathrm{in}}). \quad \text{(S9)}$$

Here, we assume $\phi_{\mathrm{in}}(\mathbf{w}_{\mathrm{in}} = 0) = 0$ since only the relative phase matters. The error term $\delta\phi_{\mathrm{in}}^{(1)}(\mathbf{w}_{\mathrm{in}})$ arises due to the presence of $\phi_{\mathrm{o}}(\mathbf{W} - \mathbf{w}_{\mathrm{in}})$ and the multiple scattering term. We apply the correction of this first estimation by multiplying $e^{-i\phi_{\mathrm{in}}^{(1)}(\mathbf{w}_{\mathrm{in}})}$ to $E(\mathbf{W}; \mathbf{w}_{\mathrm{in}})$, which converts $\phi_{\mathrm{in}}(\mathbf{w}_{\mathrm{in}})$ to $-\delta\phi_{\mathrm{in}}^{(1)}(\mathbf{w}_{\mathrm{in}})$. Once this correction is in place, we move to the next step to correct $\phi_{\mathrm{o}}(\mathbf{w}_{\mathrm{o}})$

2.3.2 Finding the approximate $\phi_{\mathrm{o}}(\mathbf{w}_{\mathrm{o}})$

We now replace $\mathbf{w}_{\mathrm{in}}$ with $\mathbf{W} = \mathbf{w}_{\mathrm{o}} + \mathbf{w}_{\mathrm{in}}$, which converts $E(\mathbf{w}_{\mathrm{o}}; \mathbf{w}_{\mathrm{in}})$ into

$$E(\mathbf{w}_{\mathrm{o}}; \mathbf{W}) = -\frac{e^{2ikz}}{\lambda^2 z^2} e^{i\phi_{\mathrm{o}}(\mathbf{w}_{\mathrm{o}})} \tilde{O}_{\mathrm{M}}(k\mathbf{W}/z) e^{-i\delta\phi_{\mathrm{in}}^{(1)}(\mathbf{W}-\mathbf{w}_{\mathrm{o}})} + E_M(\mathbf{w}_{\mathrm{o}}; \mathbf{W}) e^{-i\phi_{\mathrm{in}}^{(1)}(\mathbf{W}-\mathbf{w}_{\mathrm{o}})}. \quad \text{(S10)}$$

Similar to the correction of $\phi_{\mathrm{in}}(\mathbf{w}_{\mathrm{in}})$, we compute the angle of the inner product $\langle E(\mathbf{w}_{\mathrm{o}}; \mathbf{W}) E^*(\mathbf{w}_{\mathrm{o}} = 0; \mathbf{W})\rangle_{\mathbf{W}}$ to find the approximate $\phi_{\mathrm{o}}(\mathbf{w}_{\mathrm{o}})$:

$$\phi_{\mathrm{o}}^{(1)}(\mathbf{w}_{\mathrm{o}}) = \phi_{\mathrm{o}}(\mathbf{w}_{\mathrm{o}}) + \delta\phi_{\mathrm{o}}^{(1)}(\mathbf{w}_{\mathrm{o}}). \quad \text{(S11)}$$

Here, we assume $\phi_{\mathrm{o}}(\mathbf{w}_{\mathrm{o}} = 0) = 0$. The error term $\delta\phi_{\mathrm{o}}^{(1)}(\mathbf{w}_{\mathrm{o}})$ arises due to the residual input aberration $\delta\phi_{\mathrm{in}}^{(1)}(\mathbf{W} - \mathbf{w}_{\mathrm{o}})$ and multiple scattering term. Due to the previous correction of the input aberration, the error in the output correction is smaller than that in the input correction. After applying the output correction by multiplying $e^{-i\phi_{\mathrm{o}}^{(1)}(\mathbf{w}_{\mathrm{o}})}$ to $E(\mathbf{w}_{\mathrm{o}}; \mathbf{W})$, we go on an iteration to the input and output corrections. The iteration stops when the magnitudes of $\delta\phi_{\mathrm{in}}^{(n)}(\mathbf{w}_{\mathrm{in}})$ and $\delta\phi_{\mathrm{o}}^{(n)}(\mathbf{w}_{\mathrm{o}})$ are smaller than a certain tolerance level after n iterations. Then, the total input and output aberration corrections are respectively given as

$$\phi_{\mathrm{in}}^{\mathrm{c}}(\mathbf{w}_{\mathrm{in}}) = \sum_{j=1}^{n} \delta\phi_{\mathrm{in}}^{(j)}(\mathbf{w}_{\mathrm{in}}), \quad \text{(S12)}$$

and

$$\phi_{\mathrm{o}}^{\mathrm{c}}(\mathbf{w}_{\mathrm{o}}) = \sum_{j=1}^{n} \delta\phi_{\mathrm{o}}^{(j)}(\mathbf{w}_{\mathrm{o}}). \quad \text{(S13)}$$

2.3.3 Finding the object spectrum

With the input and output aberration corrections in place, $E(\mathbf{w}_{\mathrm{o}}; \mathbf{w}_{\mathrm{in}})$ is corrected to

$$E_{\mathrm{c}}(\mathbf{w}_{\mathrm{o}}; \mathbf{w}_{\mathrm{in}}) = -(e^{2ikz}/\lambda^2 z^2) e^{i\Delta\phi_{\mathrm{in}}(\mathbf{w}_{\mathrm{in}})} \tilde{O}_{\mathrm{M}}(k(\mathbf{w}_{\mathrm{o}} + \mathbf{w}_{\mathrm{in}})/z) e^{i\Delta\phi_{\mathrm{o}}(\mathbf{w}_{\mathrm{o}})} + E'_{\mathrm{M}}(\mathbf{w}_{\mathrm{o}}; \mathbf{w}_{\mathrm{in}}). \quad \text{(S14)}$$

Here $\Delta\phi_{\mathrm{in}}(\mathbf{w}_{\mathrm{in}}) = \phi_{\mathrm{in}}(\mathbf{w}_{\mathrm{in}}) - \phi_{\mathrm{in}}^{\mathrm{c}}(\mathbf{w}_{\mathrm{in}})$, $\Delta\phi_{\mathrm{o}}(\mathbf{w}_{\mathrm{o}}) = \phi_{\mathrm{o}}(\mathbf{w}_{\mathrm{o}}) - \phi_{\mathrm{o}}^{\mathrm{c}}(\mathbf{w}_{\mathrm{o}})$, and $E'_{\mathrm{M}}(\mathbf{w}_{\mathrm{o}}; \mathbf{w}_{\mathrm{in}}) = e^{-i\phi_{\mathrm{o}}^{\mathrm{c}}(\mathbf{w}_{\mathrm{o}})} E_{\mathrm{M}}(\mathbf{w}_{\mathrm{o}}; \mathbf{w}_{\mathrm{in}}) e^{-i\phi_{\mathrm{in}}^{\mathrm{c}}(\mathbf{w}_{\mathrm{in}})}$. We can ignore the terms $\Delta\phi_{\mathrm{in}}(\mathbf{w}_{\mathrm{in}})$ and $\Delta\phi_{\mathrm{o}}(\mathbf{w}_{\mathrm{o}})$ as they became smaller than a small tolerance level. We then replace $\mathbf{w}_{\mathrm{o}}$ with $\mathbf{W} = \mathbf{w}_{\mathrm{o}} + \mathbf{w}_{\mathrm{in}}$ in $E_{\mathrm{c}}(\mathbf{w}_{\mathrm{o}}; \mathbf{w}_{\mathrm{in}})$:

$$E_{\mathrm{c}}(\mathbf{W}; \mathbf{w}_{\mathrm{in}}) \cong -(e^{2ik}\ /\lambda^2 z^2) \tilde{O}_{\mathrm{M}}(k\mathbf{W}/z) + E'_{\mathrm{M}}(\mathbf{W}; \mathbf{w}_{\mathrm{in}}). \quad \text{(S15)}$$

The summation of $E_c(\mathbf{W};\mathbf{w}_{\text{in}})$ with respect to $\mathbf{w}_{\text{in}}$ for N orthogonal pixels leads to the object spectrum.

$$\sum_{\mathbf{w}_{\text{in}}}^{N} E_c(\mathbf{W};\mathbf{w}_{\text{in}}) \cong -\left(e^{2ikz}/\lambda^2 z^2\right) N \tilde{O}_{\text{M}}(k\mathbf{W}/z). \quad \text{(S16)}$$

The summation of $E'_{\text{M}}(\mathbf{W};\mathbf{w}_{\text{in}})$ is incoherent such that its magnitude grows with $\sqrt{N}$. Therefore, its contribution becomes smaller than the term with the object spectrum for a sufficiently large N. Taking the inverse Fourier transform of Eq. (S16) results in the object function $O_{\text{M}}(\mathbf{r})$.

2.3.4 Convergence condition

The convergence of the algorithm can be determined from the correlation of output waves between two incident wave vectors $\mathbf{w}_{\text{in}}^{(l)}$ and $\mathbf{w}_{\text{in}}^{(m)}$, which can be expressed as

$$\begin{aligned}
&\langle E\left(\mathbf{W};\mathbf{w}_{\text{in}}^{(l)}\right) E^*\left(\mathbf{W};\mathbf{w}_{\text{in}}^{(m)}\right)\rangle_{\mathbf{W}} \\
&\qquad \approx e^{i\left[\phi_{\text{in}}\left(\mathbf{w}_{\text{in}}^{(l)}\right)-\phi_{\text{in}}\left(\mathbf{w}_{\text{in}}^{(m)}\right)\right]}\alpha(z)\,\langle\left|\tilde{O}_{\text{M}}(k\mathbf{W}/\text{z})\right|^2 e^{i\left[\phi_{\text{o}}\left(\mathbf{W}-\mathbf{w}_{\text{in}}^{(l)}\right)-\phi_{\text{o}}\left(\mathbf{W}-\mathbf{w}_{\text{in}}^{(m)}\right)\right]}\rangle_{\mathbf{W}} \\
&\qquad + \langle E_{\text{M}}\left(\mathbf{W};\mathbf{w}_{\text{in}}^{(l)}\right) E_{\text{M}}^*\left(\mathbf{W};\mathbf{w}_{\text{in}}^{(m)}\right)\rangle_{\mathbf{W}}, \qquad \text{(S17)}
\end{aligned}$$

where $\alpha(z) = \left(\frac{e^{2ikz}}{\lambda^2 z^2}\right)^2$. We ignored the cross terms since they are much smaller than the second term on the right-hand side. The factor $e^{i\left[\phi_{\text{in}}\left(\mathbf{w}_{\text{in}}^{(l)}\right)-\phi_{\text{in}}\left(\mathbf{w}_{\text{in}}^{(m)}\right)\right]}$ in the first-term on the right-hand side is the input aberration that we are going to identify in this correlation, and the second term serve as a noise. The magnitude of first term are largely given by single-scattering intensity $I_S \approx \alpha(z)\left|\tilde{O}_{\text{M}}(k\mathbf{W}/\text{z})\right|^2$ and the normalized cross-correlation of output aberrations $\xi \approx \left|\langle e^{i\left[\phi_{\text{o}}\left(\mathbf{W}-\mathbf{w}_{\text{in}}^{(l)}\right)-\phi_{\text{o}}\left(\mathbf{W}-\mathbf{w}_{\text{in}}^{(m)}\right)\right]}\rangle_{\mathbf{W}}\right|/N(\mathbf{W})$, where $N(\mathbf{W})$ is the number of channel used to average over $\mathbf{W}$. Since the magnitude of ξ is related to the degree of aberration, it gets smaller as the aberration becomes complex. The magnitude of the second term is given approximately by $I_M\sqrt{N(\mathbf{W})}$. Hence, the fidelity of correction is determined by the ratio between the first and the second terms, $\chi = \xi(I_S/I_M)\sqrt{N(\mathbf{W})}$. The algorithm's convergence depends on the certain threshold value of χ that is given by single to multiple scattering intensity ratio I_S/I_M, the degree of aberration ξ, and the number of channels $N(\mathbf{W})$.

3. Data processing procedure

3.1 Reflection matrix construction with raw data

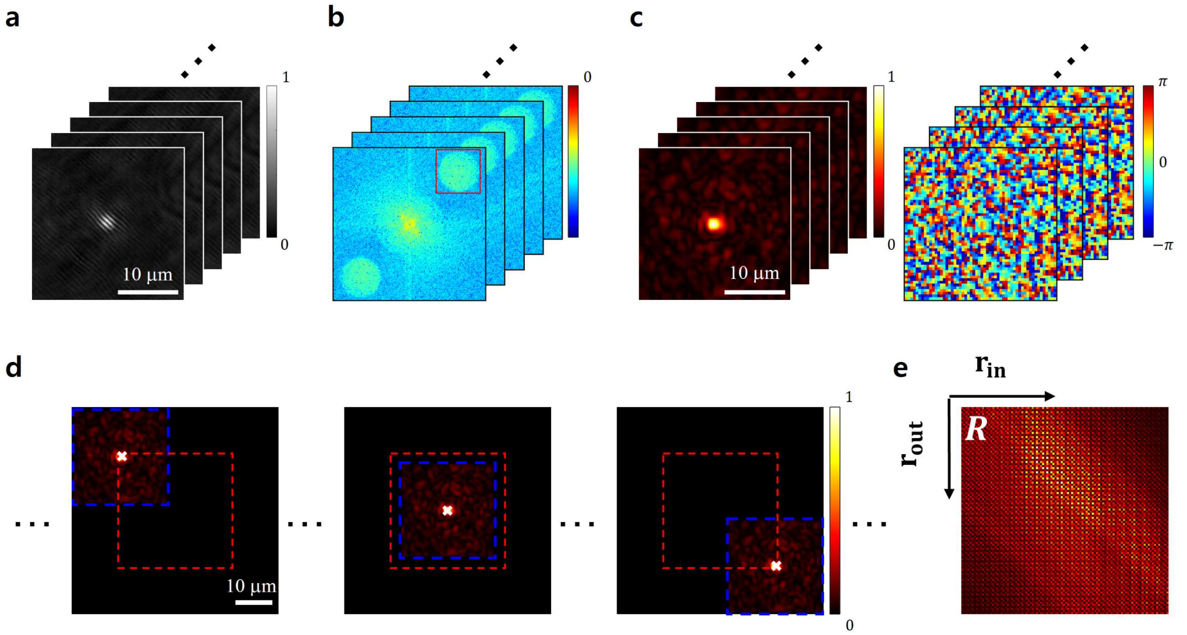

Supplementary Figure 6. Construction of reflection matrix from raw data. a, Raw interferometric images recorded at the camera for each raster scan positions of illumination. Color bar**,** normalized intensity. **b,** 2-D Fourier transforms of the raw images in **a**. The amplitudes are shown on a logarithmic scale. The red dotted boxes indicate AC components, which contain sample information. Color bar: log-scale of the normalized amplitude. **c**, Inverse Fourier transforms of the AC components in **b**, which result in the complex electric field maps. Amplitude (left) and phase (right) images are shown. Color bar: normalized amplitude (left) and phase in radians (right). **d**, Field images in the laboratory frame. The center of each complex-field map in **c** was shifted to the corresponding illumination position at the sample plane. The white × mark indicates each illumination position. The red dotted box indicates illumination ROI, and the blue dotted box is each detection region. Color bar: normalized amplitude. **e**, Reflection matrix constructed by the images in **d**. Each image in **d** was converted into column vectors and stacked in scanning order. A detailed description of raw data processing can be found in our previous work[3]

3.2 Matrix conversion and aberration correction

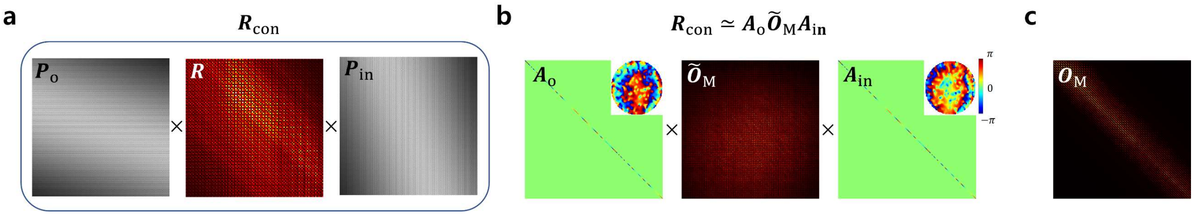

Supplementary Figure 7. Data processing procedure with reflection matrix. a, Construction of reflection matrix at conjugate plane $\boldsymbol{R}_{\mathrm{con}}$. Basis conversion from $(\mathbf{r}_{\mathrm{in}}, \mathbf{r}_{\mathrm{o}})$ to $(\mathbf{w}_{\mathrm{in}}, \mathbf{w}_{\mathrm{o}})$ is done by multiplying propagation matrix $\boldsymbol{P}_{\mathrm{in}}, \boldsymbol{P}_{\mathrm{o}}$ to the reflection matrix $\boldsymbol{R}$. Supplementary information section 2.1 explains this procedure. **b**, $\boldsymbol{R}_{\mathrm{con}}$ can be decomposed into the input matrix $\boldsymbol{A}_{\mathrm{in}}$, output aberration $\boldsymbol{A}_{\mathrm{o}}$, and the object spectrum matrix $\widetilde{\boldsymbol{O}}_{\mathrm{M}}$ after the aberration correction. The insets in $\boldsymbol{A}_{\mathrm{in}}$ and $\boldsymbol{A}_{\mathrm{o}}$ show the aberration maps composed of ϕ_{s} described in the main text. **c**, Object spectrum matrix $\boldsymbol{O}_{\mathrm{M}}$ can be obtained by 2-D inverse Fourier transform of $\widetilde{\boldsymbol{O}}_{\mathrm{M}}$. Diagonal of this matrix gives the aberration-corrected confocal image.

4. Comparison between conjugate-CLASS and pupil-CLASS

4.1 Isoplanatic patch size

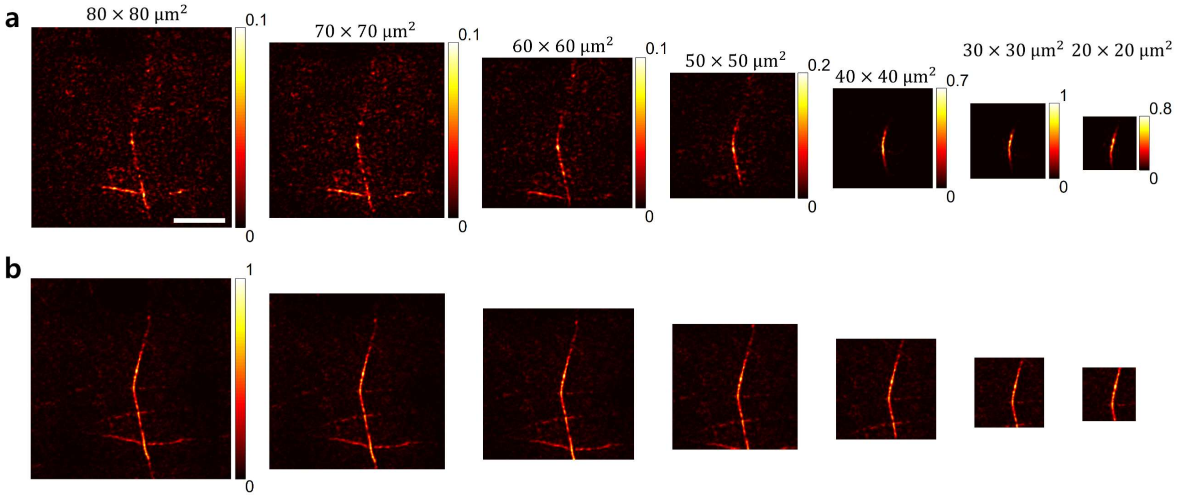

Supplementary Figure 8. Comparison of isoplanatic patch size between pupil-CLASS and conjugate-CLASS. a, Images processed by the pupil-CLASS algorithm for the different ROI sizes with respect to the same center. The size was chosen from 20 × 20 μm^2 to 80 × 80 μm^2. The signal enhancement was highest at the size of 30 × 30 μm^2, and the maximum intensity here was set to be 1. At ROIs larger than 50 × 50 μm^2, signal enhancement was reduced to 10-20 %. Furthermore, the intensity of the myelin segment was not even, and background noise was increased. At ROIs smaller than 40 × 40 μm^2, the signal enhancement was similar. However, the myelin segment was reconstructed only near the center of the ROI. From the length of the reconstructed myelin segment, the isoplanatic patch size was estimated to be 15 × 15 μm^2. The data used here was acquired at cortical layer 1 of the intact skull mouse brain. Scale bar, 20 μm. Color bars: normalized intensity with respect to the maximum value in 30 × 30 μm^2 case. **b,** Aberration-corrected image by conjugate-CLASS algorithm at ROI size of 80 × 80 μm^2 was cropped into the ROI sizes corresponding to those at **a**. Unlike pupil-CLASS, the conjugate-CLASS algorithm was able to reconstruct the myelin segment with uniform intensity and high contrast across the ROI of 80 × 80 μm^2. Color bar: normalized intensity.

4.2 Image quality

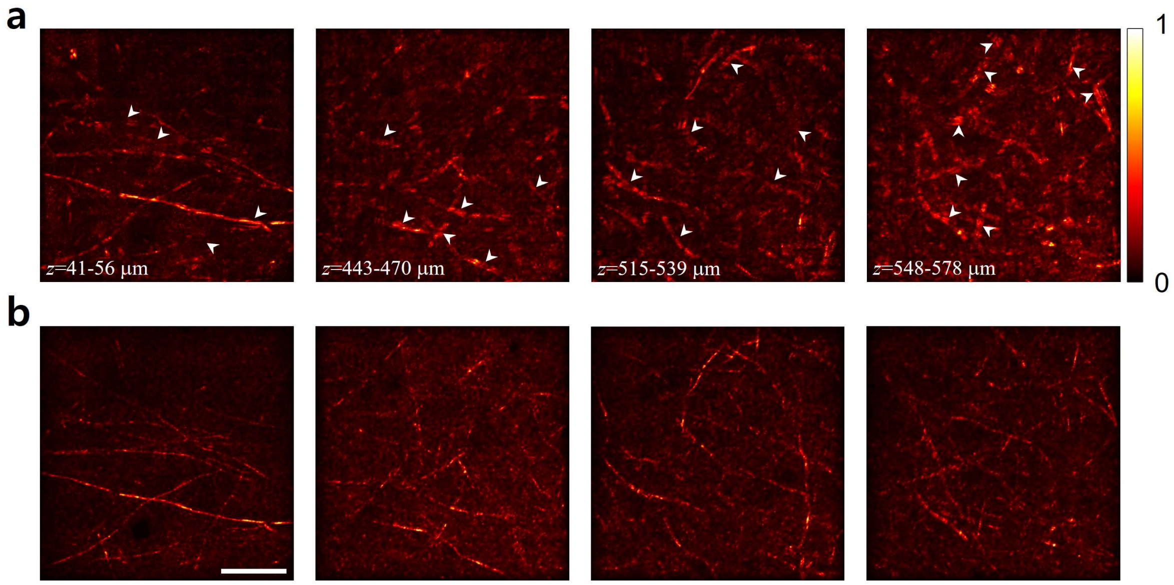

Supplementary Figure 9. Comparison of image quality between pupil-CLASS and conjugate-CLASS. a, Aberration-corrected images by the pupil-CLASS algorithm. Individual images are MIPs in the depth ranges of 41–56, 443–470, 515–539, and 548–578 μm. Each image was normalized by its maximum intensity. **b,** Aberration-corrected images by the conjugate-CLASS algorithm at the same depths as those shown in **a**. These images are the same as those in Fig. 3c in the main text. Scale bar, 40 μm. Pupil-CLASS was processed with the

patch size of $20 \times 20\ \mu m^2$, and the reconstructed images were overlapped with one another in the ratio of 50 %. Pupil-CLASS has degeneracy in the tilt and ambiguity in the defocus. (Supplementary section 4.4) This can give rise to the lateral and focal shifts of the reconstructed images. For example, the optimization can converge to the neighboring depth where there is a stronger signal than the one at the objective focus. Therefore, the same myelin segment can be reconstructed across multiple depths with different lateral shifts. This causes the blur of myelin segments in MIP images, as indicated by white arrowheads in **a**. Furthermore, the reconstruction fidelity is low when there is no myelin segment within the small patch. This is responsible for large non-uniform background noise.

4.3 Comparison of aberration maps from conjugate-CLASS and pupil-CLASS

Let us clarify the difference in the physical meaning of the aberration map between pupil-CLASS and conjugate-CLASS (Supplementary Fig. 10a). The aberration map obtained by the pupil-CLASS is the angle-dependent phase retardation. On the contrary, that obtained by the conjugate-CLASS is the local phase variations induced by the skull at the conjugate plane. Therefore, the fine phase patterns in the aberration map are associated with the fine spatial structures of the skull. The slowly varying phase pattern can be due to the slowly varying curvature of the skull. It is not legitimate to conduct the Zernike decomposition and find the defocus component directly from the conjugate-CLASS aberration map.

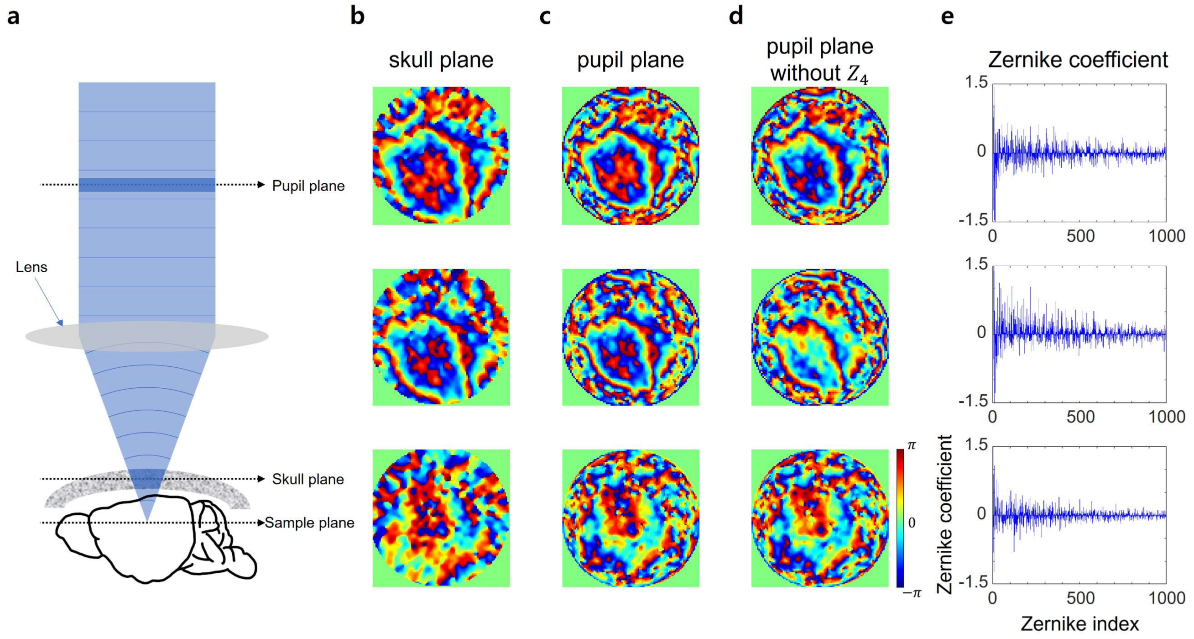

Supplementary Figure 10. Conversion of the aberration map at the skull plane to that in the pupil plane. **a,** Schematic of the wave propagation to the mouse brain through the skull. The conjugate-CLASS algorithm corrects the aberration for the dark blue region at the skull plane marked in the schematic. Therefore, the aberration map corresponds to the effective spatial phase retardation induced by the skull. On the contrary, pupil aberration marked with dark blue at the pupil plane in the schematic accounts for the angle-dependent phase retardation. **b,** conjugate-CLASS aberration maps acquired for three representative data. **c,** Pupil aberration maps obtained by converting the conjugate aberration maps in **b**. **d,** Pupil aberration maps after removing the defocus component, which is the fourth index of Zernike polynomial Z_4, in **c**. Color bar, phase in radians. **e,** Zernike coefficients of the pupil aberration in **c**. Z_4 component accounts for 0.55 %, 1.9 %, and 0.09 % of the total wavefront distortion from the top. Note that the Zernike polynomial used here follows the OSA/ANSI standard index.

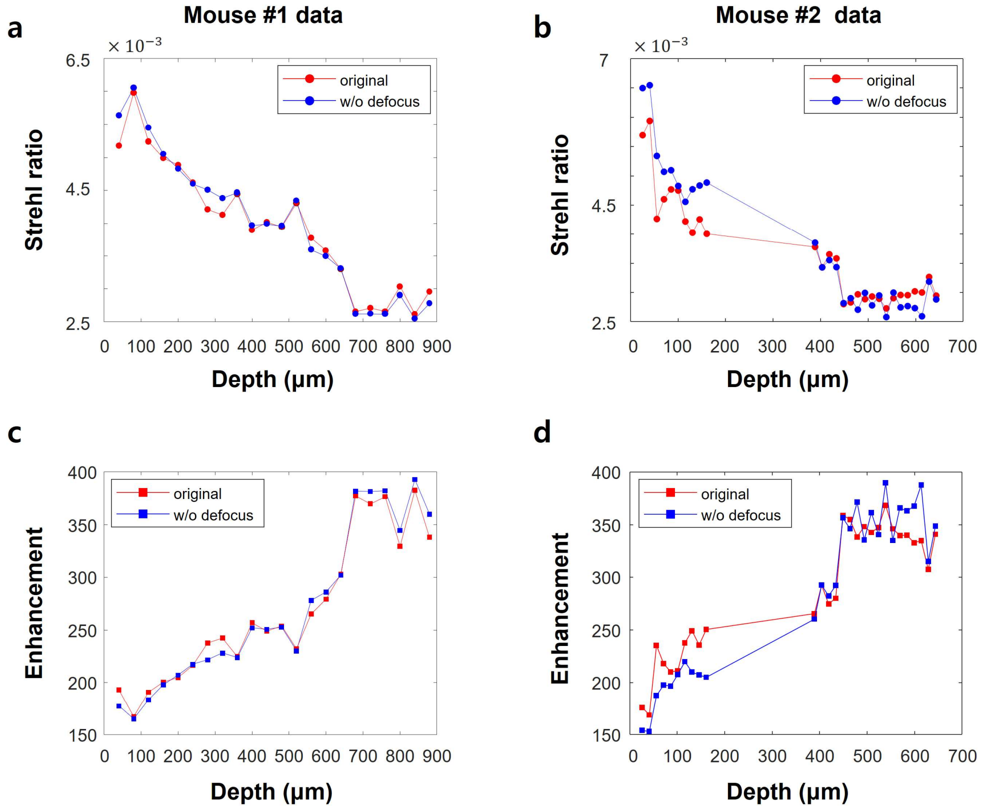

Supplementary Figure 11. Strehl ratio depending on the depth before and after Z_4 component subtraction. **a**, Strehl ratio before (red dots) and after (blue dots) Z_4 component subtraction. The data is from Supplementary Movie 3. **b,** Same as **a**, but for the data in the main text Fig. 3. For data #2, we only took data in the cortical layers 1 and 4 to focus on the myelin analysis on those layers. **c,** Expected enhancement of the PSF intensity by the aberration correction for the mouse #1 data before (red dots) and after (blue dots). Enhancement was estimated by the inverse of the Strehl ratio. **d,** Same as **c**, but for data #2.

To find the defocus component, it is necessary to convert the aberration map obtained by the conjugate-CLASS into that in the pupil plane. This is done by the following procedures. The aberration map in the conjugate plane is applied to a focused illumination at the conjugate plane. This modified wave is propagated to the object plane, and its inverse Fourier transform is taken to obtain the aberration map in the pupil plane. Supplementary Fig. 10c shows thus obtained pupil aberration map from the conjugate aberration map in Supplementary Fig. 10b. We then perform the Zernike decomposition of the pupil aberration map (Supplementary Figs. 10d and e) and found that the defocus aberration accounts for less than 2 % of the total wavefront distortion. As such, the defocus correction made little effect on the Strehl ratio and, thus, the expected enhancement estimated by the inverse of the Strehl ratio (Supplementary Fig. 11). Intuitively, the slowly varying lower-order aberration in the conjugate plane makes a negligible effect on the defocus to the spherical wave with a large spatial phase curvature converging to the object plane.

4.4 The effect of degeneracy in the tilt and ambiguity in the defocus

The original pupil-CLASS algorithm finds the aberration map that maximizes the total single scattering intensity of the reconstructed image. In this process, there can be multiple solutions that can provide the same total intensity. Suppose the aberration map has an additional phase-tilt component with respect to the ground truth map. Then, the object spectrum can have the opposite tilt component to counterbalance the tilt in the aberration map in such a way that the resulting reflection matrix is the

same as the original reflection matrix. In other words, an aberration map with an overall phase tilt causing a lateral image shift could be another solution to the algorithm.

The tilt degeneracy can cause difficulty in merging reconstructed image patches. Due to the small patch size in the pupil-CLASS, many small image patches as large as 16×16 should be merged to form an image over the full ROI. However, the unpredictable lateral image shift in each patch makes the merged image fragmented such that the reconstructed myelin fiber can be disconnected after merging. One can manually find and correct the tilt component in each aberration map. This is a substantial work by itself, and its precision cannot be guaranteed when the aberration map is too complex. The tilt degeneracy can also occur in the conjugate-CLASS algorithm, but this is not a big issue since the patch size is large enough to faithfully connect multiple patches.

In the case of defocus, it is an ambiguity rather than a degeneracy. When the myelin segments are located off from the objective focus, the algorithm tends to add a defocus in the aberration map to maximize the reconstructed image intensity. In this case, the focus of each patch can be shifted to the plane where the myelin is situated. This can also cause the fragmentation of the merged image in the pupil-CLASS especially when the myelin segment is slanted along the axial direction. The ambiguity in the defocus can also occur in the conjugate-CLASS, but the large isoplanatic patch size ensures the fidelity of merging subregion images.

5. Comparison between reflectance imaging and fluoromyelin-stained fluorescence imaging

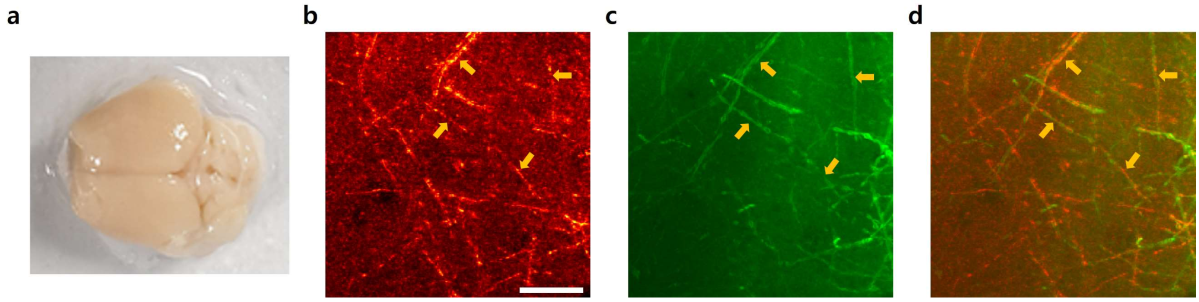

Supplementary Figure 12. Comparison between the label-free reflectance image and the fluoromyelin-stained fluorescence image. a, An ex-vivo mouse brain stained with the fluoromyelin dye (1:200 in phosphate-buffered saline, Invitrogen). **b,** Confocal reflectance image taken at cortical layer 1. Scale bar, 30 μm. **c,** Two-photon fluorescence image taken at the same region as **b**. The two-photon excitation wavelength was 1.05 μm, and the center wavelength of the emission filter was 0.63 μm. **d,** Merged image of **b** and **c**. Yellow arrows indicate the representative myelin fibers appearing in both images. This result validates that the fibrous structures in the label-free reflectance image are the myelin segments.

6. Measured myelin width before and after the conjugate-CLASS correction

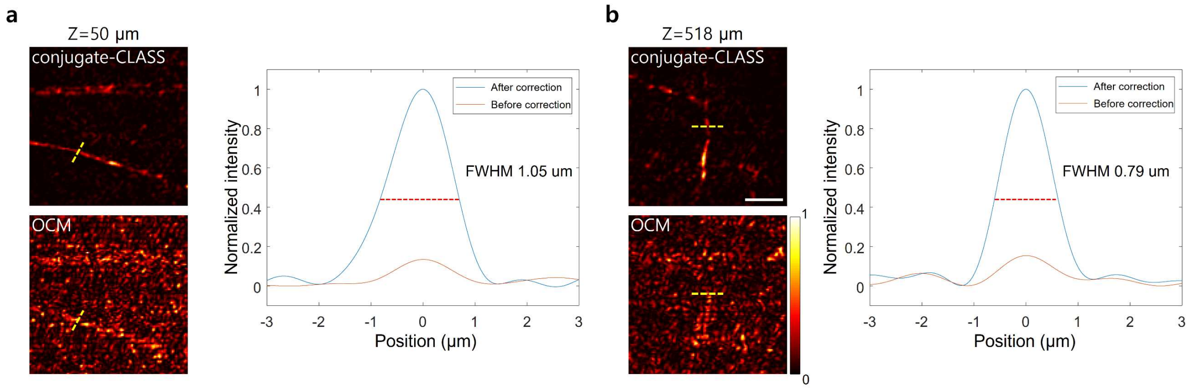

Supplementary Figure 13. Measured myelin width before and after the conjugate-CLASS correction. a, Images of myelin before and after conjugate-CLASS correction. The data was taken for a 5-week-old mouse at the depth 50 µm beneath the dura. The line profiles along the yellow dashed lines in the images are shown on the right. The full width at half maximum (FWHM) determined by the Gaussian fitting was 1.05 µm. **b,** Same as **a**, but with the data taken at the depth 518 µm beneath the dura. FWHM was 0.79 µm in this case. Scale bar, 20 µm. Color bar, normalized intensity.

References

1. Jang, M., Ruan, H., Vellekoop, I. M., Judkewitz, B., Chung, E. & Yang, C. Relation between speckle decorrelation and optical phase conjugation (OPC)-based turbidity suppression through dynamic scattering media: a study on in vivo mouse skin. *Biomed. Opt. Express* **6**, 72-85 (2015).
2. Goodman, J. W. *Introduction to Fourier Optics.* Ch.3 (W. H. Freeman, 2017).
3. Yoon, S., Lee, H., Hong, J. H., Lim, Y.-S. & Choi, W. Laser scanning reflection-matrix microscopy for aberration-free imaging through intact mouse skull. *Nat. Commun.* **11**, 5721 (2020).
