# Supplementary material for: Computational conjugate adaptive optics microscopy for longitudinal through-skull imaging of cortical myelin
Source: Nat Commun. 2023 Jan 6;14:105. doi: 10.1038/s41467-022-35738-9 (PMC9823103; doi:10.1038/s41467-022-35738-9)
Supplement: Supplementary file 3 — Description of Additional Supplementary Files [file 41467_2022_35738_MOESM3_ESM.pdf]

Description of Additional Supplementary Files

Supplementary Movie 1: 3D-rendered mouse cortex image

Supplementary Movie 2: En-face images of mouse cortex through skull #1

Supplementary Movie 3: En-face images of mouse cortex through skull #2
