# Supplementary material for: Computational conjugate adaptive optics microscopy for longitudinal through-skull imaging of cortical myelin
Source: Nat Commun. 2023 Jan 6;14:105. doi: 10.1038/s41467-022-35738-9 (PMC9823103; doi:10.1038/s41467-022-35738-9)
Supplement: Supplementary file 7 — Reporting Summary [file 41467_2022_35738_MOESM7_ESM.pdf]

nature portfolio

Corresponding author(s): Wonshik Choi
Last updated by author(s): Nov 28, 2022

Reporting Summary

Nature Portfolio wishes to improve the reproducibility of the work that we publish. This form provides structure for consistency and transparency in reporting. For further information on Nature Portfolio policies, see our Editorial Policies and the Editorial Policy Checklist.

Statistics

For all statistical analyses, confirm that the following items are present in the figure legend, table legend, main text, or Methods section.

n/a	Confirmed	
☐	☒	The exact sample size (*n*) for each experimental group/condition, given as a discrete number and unit of measurement
☐	☒	A statement on whether measurements were taken from distinct samples or whether the same sample was measured repeatedly
☒	☐	The statistical test(s) used AND whether they are one- or two-sided<br>*Only common tests should be described solely by name; describe more complex techniques in the Methods section.*
☒	☐	A description of all covariates tested
☒	☐	A description of any assumptions or corrections, such as tests of normality and adjustment for multiple comparisons
☒	☐	A full description of the statistical parameters including central tendency (e.g. means) or other basic estimates (e.g. regression coefficient) AND variation (e.g. standard deviation) or associated estimates of uncertainty (e.g. confidence intervals)
☒	☐	For null hypothesis testing, the test statistic (e.g. *F*, *t*, *r*) with confidence intervals, effect sizes, degrees of freedom and *P* value noted<br>*Give P values as exact values whenever suitable.*
☒	☐	For Bayesian analysis, information on the choice of priors and Markov chain Monte Carlo settings
☒	☐	For hierarchical and complex designs, identification of the appropriate level for tests and full reporting of outcomes
☒	☐	Estimates of effect sizes (e.g. Cohen's *d*, Pearson's *r*), indicating how they were calculated

Our web collection on statistics for biologists contains articles on many of the points above.

Software and code

Policy information about availability of computer code

Data collection	The sample stage, scanning mirrors, data acquisition board, and camera were controlled by custom Matlab (version R2018a) codes to acquire images.
Data analysis	Custom Matlab (R2018a) codes were used to develop and run the conjugate-CLASS algorithm ver1.0. All image processing including MIP and filters was performed with custom scripts written in Matlab 2018a. The analysis of myelin length was implemented by ImageJ.

For manuscripts utilizing custom algorithms or software that are central to the research but not yet described in published literature, software must be made available to editors and reviewers. We strongly encourage code deposition in a community repository (e.g. GitHub). See the Nature Portfolio guidelines for submitting code & software for further information.

Data

Policy information about availability of data

All manuscripts must include a data availability statement. This statement should provide the following information, where applicable:

- Accession codes, unique identifiers, or web links for publicly available datasets
- A description of any restrictions on data availability
- For clinical datasets or third party data, please ensure that the statement adheres to our policy

The datasets acquired for this study are available from the corresponding authors upon request. The size of the datasets is too huge to upload on the publicly accessible repository.

Human research participants

Policy information about studies involving human research participants and Sex and Gender in Research.

Reporting on sex and gender	This is not relevant to our study.
Population characteristics	This is not relevant to our study.
Recruitment	This is not relevant to our study.
Ethics oversight	This is not relevant to our study.

Note that full information on the approval of the study protocol must also be provided in the manuscript.

Field-specific reporting

Please select the one below that is the best fit for your research. If you are not sure, read the appropriate sections before making your selection.

☒ Life sciences ☐ Behavioural & social sciences ☐ Ecological, evolutionary & environmental sciences

For a reference copy of the document with all sections, see nature.com/documents/nr-reporting-summary-flat.pdf

Life sciences study design

All studies must disclose on these points even when the disclosure is negative.

Sample size	Four different mice were used for in this study. Three mice of them were used for longitudinal study. Most conclusions do not depend on the sample size. Images are usually consistent in our practice as long as the system is in proper condition.
Data exclusions	No data were excluded from the analyses.
Replication	To see if the results were reproducible, the same independent experiment was performed on other animals of similar age. For myelin imaging of cortical layer 4, all replicates with good surgery and <= 120 um skull thickness, produced similar results to those in the paper (n=3). The image qualities at all replication attempts were consistent under the imaging conditions presented in this study.
Randomization	Randomization is not relevant to this study since there are no experimental groups allocated in this study. The imaging areas of interest were randomly selected but the selected locations were repeatedly imaged for longitudinal studies.
Blinding	Not applicable, since no group was allocated. Blinding is not relevant to this study.

Reporting for specific materials, systems and methods

We require information from authors about some types of materials, experimental systems and methods used in many studies. Here, indicate whether each material, system or method listed is relevant to your study. If you are not sure if a list item applies to your research, read the appropriate section before selecting a response.

Materials & experimental systems

n/a	Involved in the study
☒	☐ Antibodies
☒	☐ Eukaryotic cell lines
☒	☐ Palaeontology and archaeology
☐	☒ Animals and other organisms
☒	☐ Clinical data
☒	☐ Dual use research of concern

Methods

n/a	Involved in the study
☒	☐ ChIP-seq
☒	☐ Flow cytometry
☒	☐ MRI-based neuroimaging

Animals and other research organisms

Policy information about studies involving animals; ARRIVE guidelines recommended for reporting animal research, and Sex and Gender in Research

Laboratory animals	Three-week old C57BL/6 mice were used for imaging until 8~10 weeks of age. Mice were maintained in temperature (20~22 'C) and

	humidity (50~55 %) controlled facilities with 12-h light/12-h dark cycles.
Wild animals	No wild animals were used in this study.
Reporting on sex	Both male and female C57BL/6 are used.
Field-collected samples	No field-collected samples were used in this study.
Ethics oversight	All the animal-related procedures were approved by the Korea University Institutional Animal Care and Use Committee (KUIACUC-2019-0024).

Note that full information on the approval of the study protocol must also be provided in the manuscript.
